# Clinical effects of polymyxin B‐immobilized fiber column direct hemoperfusion for severe bacterial meningitis: A series of 10 cases

**DOI:** 10.1002/ccr3.2756

**Published:** 2020-02-26

**Authors:** Yoko Suzuki, Shingo Kawakami, Minako Yamada, Makoto Sohmiya, Ken Shibuya, Nobuya Maeda

**Affiliations:** ^1^ Department of Neurology Omori Red Cross Hospital Tokyo Japan; ^2^ Graduate School of Health Sciences Gunma Paz University Takasaki Japan; ^3^ Department of Nephrology Omori Red Cross Hospital Tokyo Japan

**Keywords:** bacterial meningitis, disorder of consciousness, Glasgow Coma Scale, hemoperfusion

## Abstract

Our results suggest a possible role for Polymyxin B‐immobilized fiber column direct hemoperfusion in combination with standard therapy in the rapid improvement of impaired consciousness in patients with severe bacterial meningitis.

## Graphical abstract

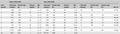

## 1 INTRODUCTION

We report 10 cases of severe bacterial meningitis with sepsis treated with PMX‐DHP in combination with the standard therapy using dexamethasone and antibiotics. Five of six patients who started PMX‐DHP within 48 hours after symptom onset had a Glasgow Outcome Scale (GOS) score of 5 at discharge.

Bacterial meningitis is an invasive disease of the central nervous system. The mortality rate depends on the causative agent but is up to 30% in high income countries.^1^, ^2^, ^3^, ^4^ The predominant causative pathogens in adults are *Streptococcus pneumoniae* and *Neisseria meningitidis*, which are responsible for 80% of all cases in Western countries.^5^ In Japan, the most common etiological agent was reported to be *S pneumoniae* (40%‐50%), followed by *Haemophilus influenzae* (about 10%).^6^ Despite the use of combination corticosteroid and antibiotic therapy, bacterial meningitis is still a major challenge to physicians. Besides high mortality, bacterial meningitis leads to a high frequency of neurological sequelae.^1^, ^4^ New treatment options are therefore urgently needed.

Direct hemoperfusion with polymyxin B‐immobilized fiber (PMX‐DHP) has been widely used to suppress systemic inflammation and to treat sepsis and septic shock.^7^, ^8^, ^9^ The PMX‐DHP column is a medical device designed to reduce circulating endotoxin produced by Gram‐negative bacteria.^10^ Several reports, however, have also noted beneficial effects of PMX‐DHP for Gram‐positive bacterial infection.^11^, ^12^ Clinical experience with PMX‐DHP for meningitis is limited though and has not been described in English‐language publications. Indeed, our first such case (case 1 in the present study) was reported in Japanese in 2013.^13^

In this study, we analyzed the clinical effects of using PMX‐DHP combined with standard dexamethasone and antibiotic therapy in 10 patients with severe bacterial meningitis, impaired consciousness, and sepsis. We also describe the first experience of the treatment we had in case 1, which was successful, for it was on this basis that we started to routinely perform PMX‐DHP in such cases and subsequently accumulated findings in the other nine cases included in this study.

## 2 SUBJECTS AND METHODS

### 2.1 Patients and diagnosis

After in the first case we treated (case 1), we started to routinely perform PMX‐DHP for patients with severe bacterial meningitis, impaired consciousness (Glasgow Coma Scale [GCS] score < 12), and systemic inflammatory response syndrome (SIRS) indicating sepsis regardless of shock. The first PMX‐DHP session is started as soon as the patient is diagnosed with severe bacterial meningitis.

In this study, we analyzed the clinical effects of PMX‐DHP for severe bacterial meningitis in a total of 10 patients treated at our institution between May 2011 and April 2018. All patients fulfilled the following inclusion criteria. (a) Bacterial meningitis diagnosed based on the following: cerebrospinal fluid (CSF) pleocytosis level (cell count > 5/µL, with a preponderance of multinuclear cells); elevated CSF pressure (>150 mmH_2_O); elevated CSF protein (>45 mg/dL); and decreased CSF glucose (<50% of serum glucose); (b) impaired consciousness defined as a GCS score < 12; (c) at least two systemic inflammatory response syndrome (SIRS) criteria.^14^

Causative agents were determined using CSF culture, blood culture, or immunochromatography for bacterial antigens in CSF and urine.

This study was carried out with the approval of the institutional ethics committee, with informed consent from each patient or their relative, and in accordance with the Declaration of Helsinki.

### 2.2 Treatment protocol

In accordance with the Japanese guidelines for the clinical management of bacterial meningitis,^6^ dexamethasone was administered before the first dose of antibiotics. The antibiotics were selected as panipenem/betamipron (PAPM/BP) and vancomycin (VCM) or meropenem trihydrate (MEPM) and VCM initially. We started PMX‐DHP as soon as the patients were diagnosed with severe bacterial meningitis regardless of the presence of shock, except for case 1.

PMX‐DHP (Toraymyxin; Toray Industries Inc.) was used with nafamostat mesilate (Torii Pharmaceutical Co.) as the anticoagulant administered at 25 mg/h for maintenance. The duration of each PMX‐DHP session was 2 hours. We performed two sessions, with the second PMX‐DHP session performed the day after the first session. Blood flow rate was maintained at 80‐120 mL/min.

### 2.3 Data collection

The following data were collected from the medical records: age, sex, causative agent, comorbidity, CSF findings, general condition (eg, SIRS, disseminated intravascular coagulation [DIC], shock, seizure, GCS score, and Sequential Organ Failure Assessment [SOFA] score), and diagnostic and follow‐up magnetic resonance imaging (MRI) findings. The outcome at discharge was graded according to the Glasgow Outcome Scale (GOS) score^15^ based on the medical records. In this study, a favorable outcome was defined as a score of 5 and an unfavorable outcome as a score of ≤4.^15^ GCS score was determined before the first PMX‐DHP session and just after the second session. SOFA score was measured just before the first PMX‐DHP session and 1 day after the second session. The central nervous system (CNS) SOFA subscore is based on the GCS score.

### 2.4 Statistical analysis

Values are expressed as the mean ± standard deviation. Differences in GCS and SOFA scores before and after PMX‐DHP were tested for significance using the paired t test. Differences in GOS scores between symptom onset and time to PMX‐DHP were tested using Fisher's exact test. The CNS SOFA subscores before and after PMX were compared using a nonparametric Wilcoxon signed‐rank test. All statistical analyses were performed using EZR (Saitama Medical Center, Jichi Medical University, Japan) with *P* values <.05 considered significant.^16^

## 3 RESULTS

### 3.1 Initial (case 1, originally reported in Japanese)

**Figure 1:**
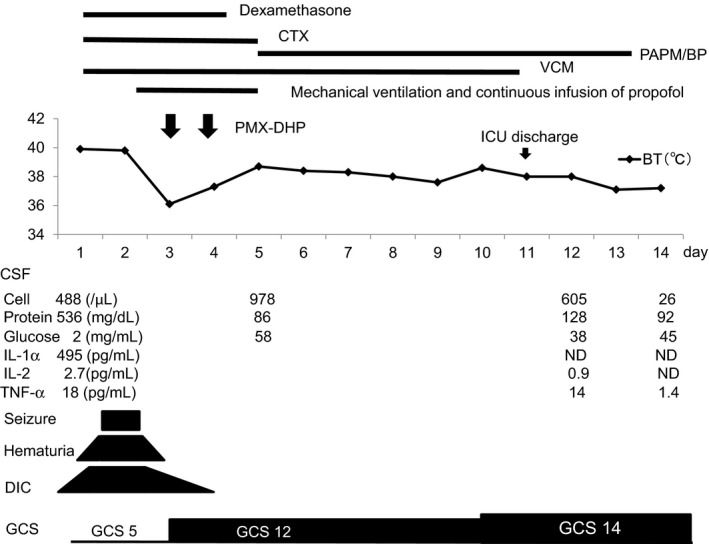
Clinical course of case 1 with bacterial meningitis. BT, body temperature; CSF, cerebrospinal fluid; CTX, Cefotaxime; DIC, disseminated intravascular coagulation; GCS, Glasgow Coma Scale; IL, interleukin; ND, not detected; PAPM/BP, panipenem/betamipron; TNF, tumor necrosis factor; VCM, vancomycin

A 61‐year‐old man presented with impaired consciousness with a GCS score of 5 (E1V1M3), SIRS (temperature, 39.6°C; leukocytes, 5800 cells/μL; pulse rate, 110/min; respiratory rate, 48/min), and elevated C‐reactive protein (8.12 mg/dL).^13^ CSF pressure was elevated at ≥625 mmH_2_O; analysis revealed pleocytosis with 480 cells/μL (78% multinuclear cells) and elevated protein at 536 mg/dL with low glucose (CSF:blood glucose ratio 0.011). Pneumococcal urine antigen test was positive, so our diagnosis was pneumococcal meningitis. *S pneumoniae* was later identified in blood culture. Corticosteroid and antibiotic therapy were started, but he developed status epilepticus and DIC on hospital day 2. Alternative treatment was urgently required to maintain life. On hospital day 3, we decided to perform PMX‐DHP to suppress the cytokine storm (Figure 1; reprinted from Suzuki et al^13^ based on the reported effectiveness of PMX‐DHP for sepsis due to Gram‐positive bacteria.^11^, ^12^ Immediately after the first PMX‐DHP session, he opened his eyes spontaneously and his consciousness level dramatically improved (Figure 1). At 54 days after admission, he was discharged fully independent in activities of daily living but with impaired memory and a GOS score of 4. He ultimately returned to work.

### 3.2 All 10 cases

Table 1 summarizes the characteristics of all 10 patients with SIRS and impaired consciousness (GCS < 12) who were diagnosed with bacterial meningitis and treated with PMX‐DHP in addition to dexamethasone and antibiotics. Mean age was 66.4 (range 40‐92) years, and SOFA and GCS scores at admission were 7.6 (range 3‐15) and 5.7 (range 3‐11), respectively. Causative agents were *S pneumoniae* in 4 cases including penicillin‐resistant *S pneumoniae* in 1 case (case 3), *Klebsiella pneumoniae* in 2 cases, methicillin‐sensitive *S aureus* in 2 cases, *Listeria monocytogenes* in 1 case, and *Group G Streptococcus* in 1 case. Table 2 shows the time to PMX‐DHP from symptom onset, GCS after PMX‐DHP, antibiotics, duration of hospitalization, neurological sequelae, MRI findings, and GOS score.

**Table 1:** Clinical and laboratory characteristics in 10 patients with bacterial meningitis

Case number	Age	Sex	Causative agents	Comorbidity	SOFA	Clinical presentation				
						SIRS	DIC	GCS (total)	Hypotension	Seizure
1	61	M	*Streptococcus pneumoniae*	Alcoholism	10	+	+	E1V1M3 (5)	−	+
2	74	F	*S pneumoniae*	‐	7	+	+	E1V1M1 (3)	−	−
3	55	M	*S pneumoniae (PRSP)*	‐	5	+	+	E1V1M1 (3)	−	+
4	40	M	*S pneumoniae*	Alcoholism, surgery for brain tumor	3	+	−	E2V3M5 (10)	−	−
5	63	M	*Listeria monocytogenes*	Alcoholism	3	+	−	E4V2M5 (11)	−	−
6	92	M	*Group G streptococcus*	Alcoholic liver cirrhosis	11	+	+	E4V1M4 (9)	+	−
7	66	F	*staphylococcus aureus (MSSA)*	IE, DM	4	+	+	E1V2M4 (7)	−	−
8	55	M	*S aureus (MSSA)*	IE	15	+	+	E1V1M1 (3)	+	−
9	86	F	*Klebsiella pneumoniae*	DM, CVD, head trauma	6	+	−	E1V1M1 (3)	−	+
10	72	M	*K pneumoniae*	Alcoholic liver cirrhosis	12	+	+	E1V1M1 (3)	−	+

Abbreviations: CVD, cardiovascular disease; DIC, disseminated intravascular coagulation; DM, diabetes mellitus; F, female; GCS, Glasgow Coma Scale; IE, infectious endocarditis; M, male; SIRS, systemic inflammatory response syndrome; SOFA, sequential organ failure assessment.

**Table 2:** Treatment and outcomes in 10 patients with bacterial meningitis

Case number	Time to PMX‐DHP from onset (hours)	GCS after post‐PMX	Antibiotics	Duration of hospitalization (d)	Neurological sequelae or outcome at discharge	MRI findings	GOS score at discharge
1	100	12	CTX 8 g + VCM 2 g PAPM/BP 4 g + VCM 1 g	54	Disturbance of memory, executive function disorder	Multiple subcortical lesions	4
2	6.5	15	PAPM/BP 4 g + VCM 1.5 g	19	Free from neurological sequelae		5
3	19.5	12 (Day 3)	PAPM/BP 4 g	38	Slight disturbance of memory	Left temporal, parietooccipital lesion, ventriculitis	5
4	6.5	13	PAPM/BP 4 g + VCM 3 g PAPM/BP 4 g CTX 4 g	34	Slight attention deficit	Ventriculitis	5
5	42	15	PAPM/BP 4 g + VCM 6 g ABPC 12 g	28	Free from neurological sequelae		5
6	26.5	13	MEPM 4 g + VCM 0.5 g SBT/ABPC 9 g SBT/ABPC 9 g + CLDM 2.4 g + LVFX 0.5 g CLDM 2.4 g + LVFX 0.25 g MEPM 1.5 g + CLDM 2.4 g + LVFX 0.25 g MEPM 1.5 g + LVFX 0.25 g LVFX 0.25 g MEPM 4 g + LVFX 0.25 g MEPM 4 g ABPC 6 g MEPM 4 g	104	Disuse syndrome	Ventriculitis, small infarction in left frontal lobe, lumbar discitis, abscesses in bilateral psoas muscles	5
7	142	15 (Day 7)	PAPM/BP 4 g PAPM/BP 4 g + VCM 1.5 g SBT/ABPC 3 g + VCM 1.5 g SBT/ABPC 6 g	56	Loss calculation, Lt. hemiparesis	Multiple infarctions in right ACA + MCA area	4
8	63	3	MEPM 8 g + VCM 1 g	29	Death	Multiple infarctions in bilateral thalamus, midbrain, temporal, frontal lobes, and cerebellum	1
9	41.5	12 (Day 5)	MEPM 6 g MEPM 6 g + CLDM 1.8 g	154	Dementia, dysphagia, disuse syndrome	Multiple subarachnoid abscesses, ventriculitis, multiple white matter lesions in right frontal lobe, right occipital lobe, and cerebellum	3
10	55	3	MEPM 6 g MEPM 6 g + LVFX 0.25 g	21	Death	Multiple infarctions in bilateral thalamus, midbrain, cerebellum, and cerebrum, ventriculitis, multiple subarachnoid abscesses	1

Note

Day 3, Day 5, and Day 7 refer to the 3rd, 5th, and 7th day after the first session of PMX‐DHP, respectively.

Abbreviations: ACA, anterior cerebral artery; GOS, Glasgow Outcome Scale; MCA, middle cerebral artery; MRI, magnetic resonance imaging; PMX‐DHP, direct hemoperfusion with polymyxin B‐immobilized fiber.

**Figure 2:**
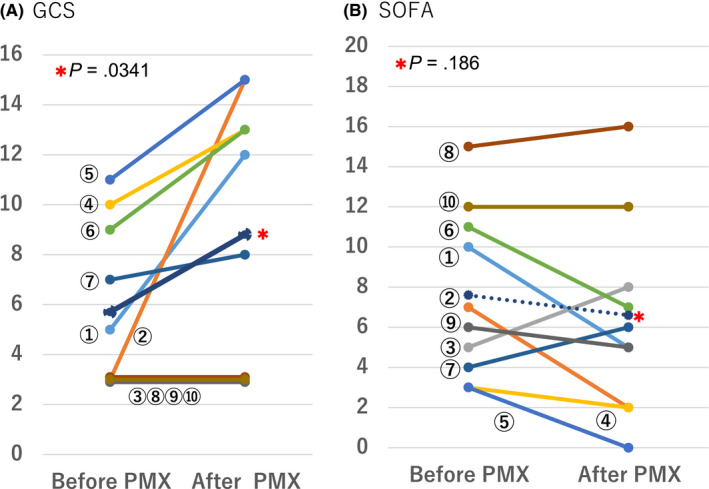
Changes in GCS (A) and SOFA scores (B) in 10 patients with bacterial meningitis in this study. The scores were determined just before the first session of PMX‐DHP (before PMX‐DHP) and just after the second session (after PMX‐DHP) in the case of the GCS and at 1 day after in the case of the SOFA. The number in parentheses is the number of cases. The dotted line is the mean. GCS, Glasgow Coma Scale; SOFA, sequential organ failure assessment

Glasgow Coma Scale score was improved by day 7 after the first PMX‐DHP session in 8 of 10 patients (Table 2). Two patients (cases 8 and 10) showed no change in GCS score and ultimately did not survive. In 6 of 10 patients (cases 1, 2, 4‐7), GCS score had improved by the end of the second PMX‐DHP session. Mean GCS score of the 10 patients was significantly improved from 5.7 ± 3.3 before the first PMX‐DHP session to 8.8 ± 5.3 at the end of the second session (*P* = .0341), as shown in Figure 2A. Mean SOFA score decreased from 7.6 ± 4.2 before PMX‐DHP to 6.3 ± 4.8 within 24 hours after PMX‐DHP (Figure 2B), but there was no significant difference (*P* = .186). The CNS SOFA subscore was 3.4 ± 0.84 before the first PMX‐DHP and 2.3 ± 1.7 at the end of the second PMX‐DHP session (*P* = .0545).

Table 3 shows the CSF findings before and after PMX‐DHP in 10 cases. Nine cases (not including case 10) showed improvement in CSF findings. Cases 1 and 7, in which repeat CSF analysis was performed relatively early after PMX‐DHP (first and second day after the second session of PMX‐DHP), once showed an increase in cell count and then a decrease.

**Table 3:** CSF findings before and after PMX‐DHP in 10 patients with bacterial meningitis

Case number	Before PMX‐DHP				After PMX‐DHP							
	Cell count (cells/μL)	Protein (mg/dL)	Glucose (mg/dL)	Day (d)	Cell count (cells/μL)	Protein (mg/dL)	Glucose (mg/dL)	Day (d)	Cell count (cells/μL)	Protein (mg/dL)	Glucose (mg/dL)	Day (d)
1	480	536	2	1 (−3)	978	86	58	5 (1)	26	92	45	14 (10)
2	289	138	47	1 (−1)	27	43	49	6 (4)				
3	362	682	0	1 (−1)	342	366	32	8 (6)	101	183	50	16 (14)
4	1173	332	0	1 (−1)	135	42	57	5 (3)	20	57	42	20 (18)
5	471	368	50	1 (−2)	128	118	50	7 (4)	59	60	53	21 (18)
6	1407	590	1	1 (−1)	211	613	28	5 (3)	< 1	108	41	12 (10)
7	151	115	58	7 (−1)	2208	304	46	10 (2)	12	49	45	28 (20)
8	1367	56	41	1 (−1)	2	63	63	8 (6)				
9	17 152	1028	1	3 (−1)	1680	801	10	8 (4)	53	197	107	72 (68)
10	4437	1213	1	3 (−1)	197 333	1580	7	6 (2)				

Note

Day is hospital day and (d) is days from second session of PMX‐DHP.

Abbreviations: CSF, cerebrospinal fluid; PMX‐DHP, direct hemoperfusion with polymyxin B‐immobilized fiber.

**Figure 3:**
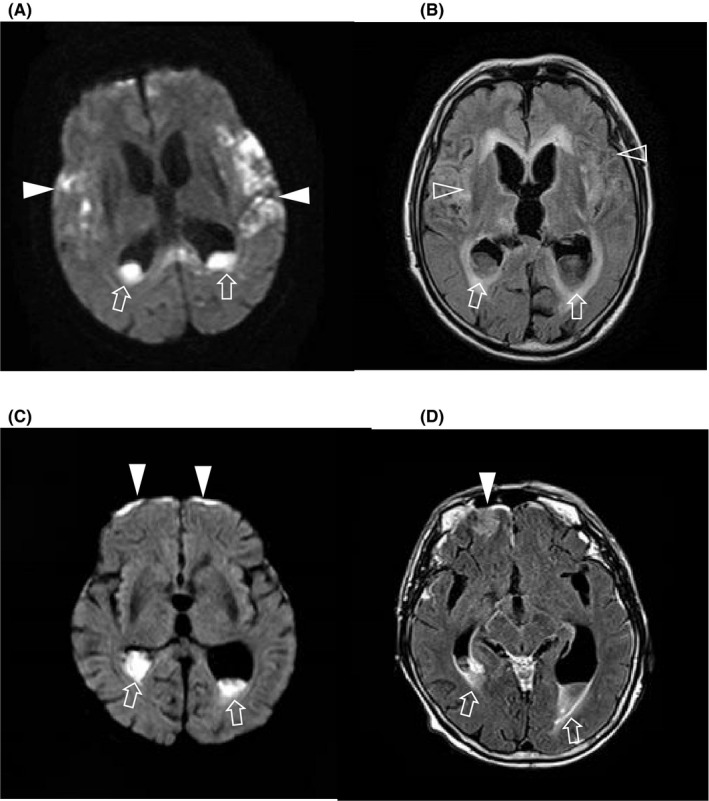
Brain MRI diffusion‐weighted image (A) and FLAIR image (B) in case 10 presenting with subarachnoid abscess (arrowheads) and ventriculitis (arrows) on hospital day 12. Brain MRI diffusion‐weighted image (C) and FLAIR image (D) in case 9 presenting with subarachnoid abscess (arrowheads) and ventriculitis (arrows) on hospital day 29

Five patients had favorable outcomes with GOS scores of 5, and the other 5 patients had unfavorable outcomes with GOS scores of ≤4. Cases 9 and 10 had suspected brain abscess and were treated with levofloxacin or clindamycin in addition to MEPM. An MRI diffusion‐weighted image (Figure 3A) and a FLAIR image (Figure 3B) in case 10 revealed subarachnoid abscess and ventriculitis on hospital day 12. MRI findings of case 9 on day 29 (Figure 3C,D) revealed subarachnoid abscess and ventriculitis. Although this patient was hospitalized for 154 days at our institution, she survived and was transferred to a long‐term medical care facility because of the need for considerable assistance with activities of daily living due to worsened dementia, dysphagia, and disuse syndrome. These two cases with subarachnoid abscess and ventriculitis were refractory to treatment. Case 6 was also found to have lumbar disk abscess and bilateral psoas muscle abscess; therefore, we could not deescalate the antibiotics. He was hospitalized for more than 100 days but discharged with favorable outcome.

Case 8 did not survive, and the cause of death was possibly infectious endocarditis; the bacterial meningitis had actually improved, evidenced by the decreased CSF cell count to 2 cells/μL on hospital day 8.

Five patients (cases 2‐6) of the six patients who started PMX‐DHP within 48 hours after symptom onset showed GOS scores of 5 at discharge. However, none of the four patients who started PMX‐DHP after 48 hours had a GOS score of 5 at discharge. An earlier start of PMX‐DHP yielded significantly favorable outcomes compared to a late start (*P* = .0476) (Table 4).

**Table 4:** Time from onset to PMX‐DHP and GOS score at discharge

Time to PMX‐DHP	GOS score at discharge		Total cases
	5	1‐4	
≤48 h	5 cases (83.3%)	1 case (16.7%)	6 cases
>48 h	0 cases (0%)	4 cases (100%)	4 cases

Note

Fisher's exact test, *P* = .0476.

Abbreviations: GOS, Glasgow Outcome Scale; PMX‐DHP, direct hemoperfusion with polymyxin B‐immobilized fiber.

## 4 DISCUSSION

In this study, we retrospectively analyzed 10 patients with severe bacterial meningitis treated with PMX‐DHP in addition to dexamethasone and antibiotics. Of the 10 patients, 8 survived, 5 of whom had favorable outcomes. To our knowledge, this is the first study to report the improvement of severe bacterial meningitis with PMX‐DHP in combination with conventional dexamethasone and antibiotic therapy.

PMX‐DHP is widely used for treating sepsis caused by Gram‐negative bacteria, such as in urinary tract infection and intraperitoneal (intra‐abdominal) infection.^7^ The difference between treating these infections and bacterial meningitis is the dose of antibiotics. Most antibiotics have a poor penetration from blood to CSF due to the blood‐brain barrier, and therefore, larger doses of antibiotics are required for treating bacterial meningitis than other infections.

### 4.1 Rapid improvement of impaired consciousness

Treatment resulted in dramatic improvement of impaired consciousness rapidly (Figure 2A). SOFA score, however, showed no immediate significant improvement (Figure 2B). A possible reason is that PMX‐DHP tends to deplete platelets and increase SOFA score for several days.

### 4.2 Clinical effect on *S pneumoniae*

*Streptococcus pneumoniae* is the most common causative agent of bacterial meningitis.^5^, ^6^, ^17^, ^18^ Previous studies have reported mortality rates of patients with pneumococcal meningitis as 14% (n = 58),^4^ 20% (n = 310),^18^ and 24% (n = 87).^19^ In our study, four patients had pneumococcal meningitis and all four survived, which is considerably better than in previous reports. Also, de Gans et al^4^ showed that 15 of 58 (26%) patients with pneumococcal meningitis treated with conventional therapy had GOS scores of 1‐4 at 8 weeks after admission. In our study, 3 of 4 patients had a GOS score of 5, with the remaining one patient having a GOS score of 4 at discharge. Our results can be regarded as favorable with respect to those obtained by de Gans et al.

### 4.3 Mechanism of effect of PMX‐DHP in bacterial meningitis

The mechanism remains uncertain at this time. In the pathophysiology of bacterial meningitis, bacterial components such as lipopolysaccharide (LPS, endotoxin), lipoteichoic acid (LTA), and peptidoglycan induce tumor necrosis factor (TNF)‐α and interleukin (IL)‐1, leading to leukocyte activation, vascular endothelial damage, and coagulation activation through the cytokine and arachidonic acid cascades.^2^ When this inflammation spreads to the brain parenchyma and cerebral blood vessels, it causes cerebral edema, increased intracranial pressure, cerebrovascular disability, cerebral vasculitis, and neuronal damage, which leads to poor outcomes such as neurological sequelae and death.^2^ PMX‐DHP may suppress inflammation by absorbing activated leukocytes^20^ and LTA^21^ and reducing pro‐inflammatory cytokines.^22^, ^23^ Eight of our 10 patients were identified to have Gram‐positive bacteria, which do not have lipopolysaccharide. However, PMX fibers were reported to adsorb LTA,^21^ and PMX‐DHP is effective for septic shock due to Gram‐positive bacteria.^11^, ^12^

The theory of cytokinesis proposes that removal of cytokines and endotoxins by blood purification therapy results in improvement of cell function. This may cause polymorphonuclear leukocyte relocation and monocyte migration to the infection site.^24^ Cases 1 and 7, for example, where repeat CSF analysis was performed relatively early after PMX‐DHP, initially showed an increase in cell count. Relocation of leukocytes may occur immediately after PMX‐DHP.

### 4.4 Starting PMX‐DHP early was associated with favorable outcomes

Five of our 10 patients had unfavorable outcomes (GOS score ≤ 4) even though PMX‐DHP was used in addition to steroid and antibiotic therapy. There are two possible reasons for this. The first is a delay in starting PMX‐DHP, because cytokines are released within several hours of the onset of bacterial meningitis and increase rapidly in CSF in animal models^25^ and humans.^26^ Also, early treatment with dexamethasone was associated with reduced risk of unfavorable outcomes.^4^ As with corticosteroid therapy, PMX‐DHP should be performed as early as possible. We attempted to start PMX‐DHP within 48 hours of the onset of meningitis, and 5 of the 6 patients for whom this was started within 48 hours had favorable outcomes, with a GOS score of 5. However, none of the four patients in the delayed group had favorable outcomes (Table 3). Thus, starting PMX‐DHP early may be associated with favorable outcome. Indeed, the timing of PMX‐DHP initiation is reported to be an important factor for survival in patients with sepsis.^27^, ^28^ We recommend that PMX‐DHP be performed as soon as possible for bacterial meningitis with impaired consciousness (GCS score < 12).

There are several reasons why PMX‐DHP was not performed within 48 hours in these four patients. Case 1 was the first case, and the decision to perform PMX‐DHP took some time. Case 7 was referred to our department only after lengthy treatment (6 days) in other departments. Case 8 was referred to our department from another hospital after 2 days. Case 10 had alcoholic cirrhosis and hyperammonemia, and bacterial meningitis was diagnosed only after treatment of hepatic encephalopathy.

The second possible reason for unfavorable outcomes is ventriculitis. Ventriculitis is associated with refractory meningitis, with a mortality rate of 40%‐80%.^29^ There were five patients with ventriculitis in our study (cases 3, 4, 6, 9, and 10). The three patients with non‐*K pneumoniae* infection (cases 3, 4, and 6) were discharged with favorable outcomes, whereas both patients with *K pneumoniae* infection (cases 9 and 10) had unfavorable outcomes possibly due to infection with hypervirulent (hypermucoviscous) *K pneumoniae*.^30^

A standard PMX‐DHP procedure comprises two sessions of 2 hours each. Prolonging the duration and/or performing more than two sessions may improve the outcome.^31^

### 4.5 Limitations

There are several limitations of this study. First, the number of patients was small, which limits the generalization of the results. Second, there was no control group; this made it difficult to statically evaluate the true effects of the improvement in impaired consciousness. Third, we did not measure endotoxin levels and thus could not clarify the mechanism involved in the improvement of impaired consciousness. We intend to address these limitations in future research.

## 5 CONCLUSION

To our knowledge, this is the first study to report the successful use of PMX‐DHP alongside conventional steroid and antibiotic therapy for bacterial meningitis. Bacterial meningitis is a neurological emergency. Our results suggest a possible role for PMX‐DHP in combination with standard therapy in the rapid improvement of consciousness in patients with severe bacterial meningitis. Accordingly, we suggest that PMX‐DHP be started at the earliest possible opportunity in combination with conventional steroid plus antibiotic therapy for the treatment of severe bacterial meningitis presenting with sepsis regardless of shock and with impaired consciousness (GCS score < 12). Further large‐scale studies are required to confirm the clinical effectiveness.

## CONFLICT OF INTEREST

None declared.

## AUTHOR CONTRIBUTION

YS: involved in research concept and design, data analysis and interpretation, and writing the article. YS, SK, MY, KS, and NM: involved in collection and/or assembly data. YS and MS: involved in critical revision of the article. YS, SK, MY, MS, KS, and NM: involved in final approval of article.

## Data Availability

The datasets obtained and/or analyzed during the current study are available from the corresponding author upon reasonable request.
